# Resolving the puzzle of single-atom silver dispersion on nanosized γ-Al_2_O_3_ surface for high catalytic performance

**DOI:** 10.1038/s41467-019-13937-1

**Published:** 2020-01-27

**Authors:** Fei Wang, Jinzhu Ma, Shaohui Xin, Qiang Wang, Jun Xu, Changbin Zhang, Hong He, Xiao Cheng Zeng

**Affiliations:** 1 State Key Joint Laboratory of Environment Simulation and Pollution Control, Research Center for Eco-Environmental Sciences, Chinese Academy of Sciences, Beijing, 100085 China; 2 University of Chinese Academy of Sciences, Beijing, 100049 China; 3 Center for Excellence in Regional Atmospheric Environment, Institute of Urban Environment, Chinese Academy of Sciences, Xiamen, 361021 China; 4 State Key Laboratory of Magnetic Resonance and Atomic and Molecular Physics, Wuhan Institute of Physics and Mathematics, Chinese Academy of Sciences, Wuhan, 430071 China; 5 Department of Chemistry, Department of Chemical & Biomolecular Engineering, and Department of Mechanical & Materials Engineering, University of Nebraska-Lincoln, Lincoln, NE 68588 USA

**Keywords:** Catalytic mechanisms, Heterogeneous catalysis, Surface spectroscopy

## Abstract

Ag/γ-Al_2_O_3_ is widely used for catalyzing various reactions, and its performance depends on the valence state, morphology and dispersion of Ag species. However, detailed anchoring mechanism of Ag species on γ-Al_2_O_3_ remains largely unknown. Herein, we reveal that the terminal hydroxyls on γ-Al_2_O_3_ are responsible for anchoring Ag species. The abundant terminal hydroxyls existed on nanosized γ-Al_2_O_3_ can lead to single-atom silver dispersion, thereby resulting in markedly enhanced performance than the Ag cluster on microsized γ-Al_2_O_3_. Density-functional-theory calculations confirm that Ag atom is mainly anchored by the terminal hydroxyls on (100) surface, forming a staple-like local structure with each Ag atom bonded with two or three terminal hydroxyls. Our finding resolves the puzzle on why the single-atom silver dispersion can be spontaneously achieved only on nanosized γ-Al_2_O_3_, but not on microsized γ-Al_2_O_3_. The obtained insight into the Ag species dispersion will benefit future design of more efficient supported Ag catalysts.

## Abstract (web summary)

Detailed atom-level anchoring mechanism of Ag species on γ-Al_2_O_3_ is largely unknown for the widely used Ag/γ-Al_2_O_3_ catalyst. Here, the authors demonstrate that single-Ag atom can be only anchored by the terminal hydroxyls on the (100) surfaces of γ-Al_2_O_3_ through consuming two or three terminal hydroxyls.

## Introduction

Alumina-supported silver (Ag/Al_2_O_3_) is a widely used catalyst in a variety of industrial applications, such as NO_*x*_ emission control via selective catalytic reduction (SCR) with using hydrocarbons (HC-SCR)^1–6^ or NH_3_ (NH_3_-SCR)^7,8^, soot oxidation^9,10^, selective catalytic oxidation of ammonia (NH_3_–SCO)^11–13^, oxidation of volatile organic compounds^14,15^, ethylene epoxidation^16,17^, among others. The valence state, dispersion, and morphology of Ag species have been found to play important roles to the activities of Ag-based catalysts as these factors are all closely related to how Ag species are anchored on the Al_2_O_3_ surface. Hence, it is of both fundamental and technical importance to uncover the anchoring mechanism of Ag species on Al_2_O_3_ for the design of more efficient Ag/Al_2_O_3_ catalysts.

The strong metal–support interaction has been often identified as a key factor to affecting metal anchoring on oxide surfaces^18–20^. Both previous experiments^21^ and DFT calculations^22^ have shown that the presence of electronic defects on reducible oxides (e.g., CeO_2_ and TiO_2_) is the direct cause for the strong interaction between metal and support^19,23^. Due to the lack of electronic defects on nonreducible oxides such as Al_2_O_3_ and SiO_2_, some other factors must be responsible for the dispersion and thermal stability of active metals. Kwak et al.^23^ reported that unsaturated pentacoordinate Al^3+^ (Al^3+^_penta_) centers on the γ-Al_2_O_3_ surface, created by dehydration and dehydroxylation at 573 K, are the anchoring sites for Pt to entail strong interactions with γ-Al_2_O_3_ through oxygen bridges. They proposed that the coordinative saturation of Al^3+^_penta_ sites is the driving force for Pt anchoring on γ-Al_2_O_3_. However, the source of the oxygen bridges was not explained specifically. Surface OH groups have also been shown to influence the dispersion of active metals on support (η-Al_2_O_3_ and SiO_2_)^24,25^, while the interaction between metals and OH groups has not been elucidated at the atomic level. Despite Ag/γ-Al_2_O_3_ catalysts have been extensively investigated, particularly on their valence state, dispersion, or morphology of Ag species, detailed anchoring mechanism of the Ag species on γ-Al_2_O_3_ surface is still unclear to date. Hence, it is timely to explore whether the Al^3+^_penta_ sites, OH groups, or other sites are responsible for anchoring Ag species on γ-Al_2_O_3_, as well as the associated chemical mechanism for single-atom Ag dispersion.

Here, we select two kinds of γ-Al_2_O_3_, nanosized γ-Al_2_O_3_ and microsized γ-Al_2_O_3_ (details given in Supplementary Information Methods), as the support for the preparation of Ag/Al_2_O_3_ catalysts. Most surprisingly, we observe that Ag can be atomically dispersed on the nanosized γ-Al_2_O_3_, whereas Ag mainly exits in the form of nanoclusters on the microsized γ-Al_2_O_3_. By using a series of surface-science measurements and Density-functional-theory (DFT) computation, we reveal that the terminal hydroxyl groups on γ-Al_2_O_3_ surface are responsible for anchoring the Ag species on γ-Al_2_O_3_. More importantly, we find that the presence of abundant terminal hydroxyl groups on the nanosized γ-Al_2_O_3_ leads to the single-atom dispersion of Ag species on the γ-Al_2_O_3_ surface. As expected, the Ag/Al_2_O_3_ with single-atom dispersion of Ag species demonstrates markedly higher performance than cluster-dispersion of Ag species for the SCR of NO (SCR-NO) reaction.

## Results

### Ag dispersion on nanosized γ-Al_2_O_3_ and microsized γ-Al_2_O_3_

**Fig. 1:**
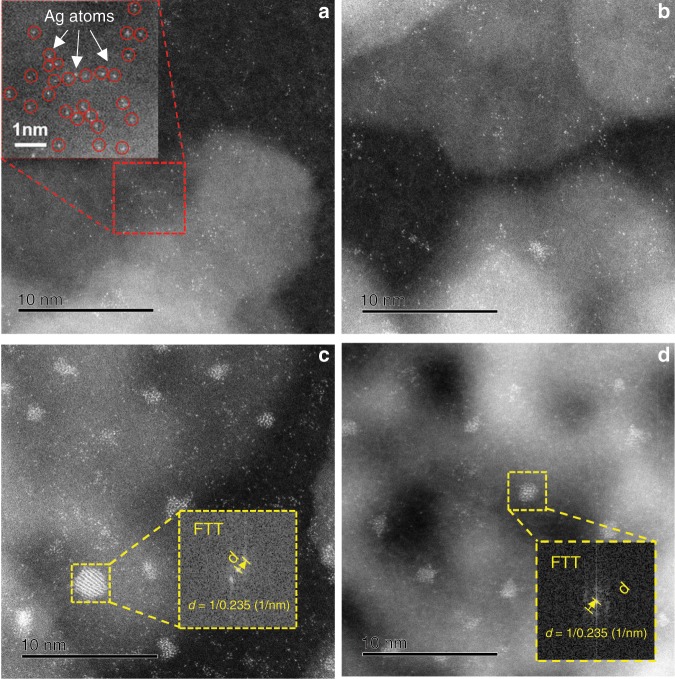
HAADF-STEM images of Ag/γ-Al_2_O_3_ samples. **a** 1% Ag/nano-Al_2_O_3_, **b** 2% Ag/nano-Al_2_O_3_, **c** 4% Ag/nano-Al_2_O_3_ and **d** 1% Ag/micro-Al_2_O_3_.

Nanosized γ-Al_2_O_3_ (nano-Al_2_O_3_, average size: 10 nm) and microsized γ-Al_2_O_3_ (micro-Al_2_O_3_, average size: 5 μm) were employed as the supports for the preparation of Ag/Al_2_O_3_ catalysts. HAADF-STEM images were taken to reveal the Ag morphology and dispersion on Ag/nano-Al_2_O_3_ with different Ag loadings (1, 2, 4%) and 1% Ag/micro-Al_2_O_3_ (see Fig. 1). As shown in (Fig. 1a, b), atomically dispersed Ag species were observed for the 1% Ag/nano-Al_2_O_3_ sample, and Ag species still maintained atomic dispersion while a very few sub-nanometer Ag cluster species were also present on 2% Ag/nano-Al_2_O_3_. When the Ag loading was increased up to 4% (Fig. 1c), more Ag clusters appeared alongside Ag atoms, and the lattice spacing of Ag clusters was 0.235 nm, corresponding to the (111) planes of cubic metallic Ag (JCPDS file no. 01-071-3762)^26–28^. In contrast, as presented in Fig. 1d, single-atom Ag was rarely observed on 1% Ag/micro-Al_2_O_3_, while Ag nanoparticles of 2 nm average size (with lattice spacing = 0.235 nm) comprised the main morphology for Ag species. These results indicate that 1% Ag can be atomically dispersed on nano-Al_2_O_3_, whereas the Ag species agglomerates into nanoparticles on micro-Al_2_O_3_.

### HC-SCR activity of Ag/Al_2_O_3_ with atomically dispersed Ag

**Fig. 2:**
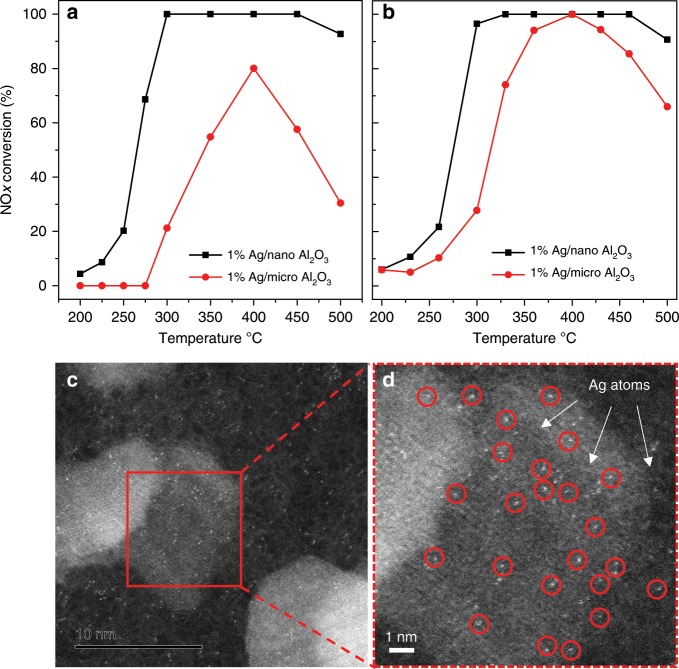
HC-SCR activities of 1% Ag/γ-Al_2_O_3_ and HAADF-STEM images of 1% Ag/nano-Al_2_O_3_ after testing. NO_*x*_ conversion for **a** C_3_H_6_–H_2_-SCR (NO 800 ppm, C_3_H_6_ 1565 ppm, H_2_ 1%, O_2_ 10%, N_2_ balance. GHSV 100,000 h^−1^), **b** ethanol-SCR (NO 800 ppm, C_2_H_5_OH 1565 ppm, O_2_ 10%, H_2_O 5%, N_2_ balance. GHSV 100,000 h^−1^) of NO over 1% Ag/nano-Al_2_O_3_ and 1% Ag/micro-Al_2_O_3_. **c**, **d** HAADF-STEM image and drawing of partial enlargement of 1% Ag/nano-Al_2_O_3_ after ethanol-SCR reaction.

Ag/Al_2_O_3_ catalysts have been extensively explored for the SCR of NO reaction, where both isolated Ag^+^ species and Ag_n_^δ+^ clusters are generally considered as the active centers^1,29–31^. We tested the 1% Ag/nano-Al_2_O_3_ and 1% Ag/micro-Al_2_O_3_ catalysts for C_3_H_6_-H_2_-SCR and ethanol-SCR of NO to investigate the effect of Ag dispersion on catalytic activity. The results are shown in Fig. 2a, b, respectively. The 1% Ag/nano-Al_2_O_3_ presented much higher activity than 1% Ag/micro-Al_2_O_3_ for both C_3_H_6_-H_2_-SCR and ethanol-SCR, demonstrating that the atomically dispersed Ag species on Ag/nano-Al_2_O_3_ are markedly superior to the Ag nanoparticles on Ag/micro-Al_2_O_3_. These results suggest that isolated Ag^+^ species are more active than Ag_n_^δ+^ clusters or Ag nanoparticles for NO_x_ reduction. After HC-SCR of NO testing, the morphology of single-atom dispersed Ag on 1% Ag/nano-Al_2_O_3_ catalyst was investigated again by using HAADF-STEM, and the images are shown in Fig. 2c, d. There was no sign of agglomeration of single-atom Ag species on spent Ag/nano-Al_2_O_3_ sample, indicating the single-atom dispersed Ag on the nano-Al_2_O_3_ catalyst was very stable.

### Charge state of Ag species and its connection with γ-Al_2_O_3_

**Fig. 3:**
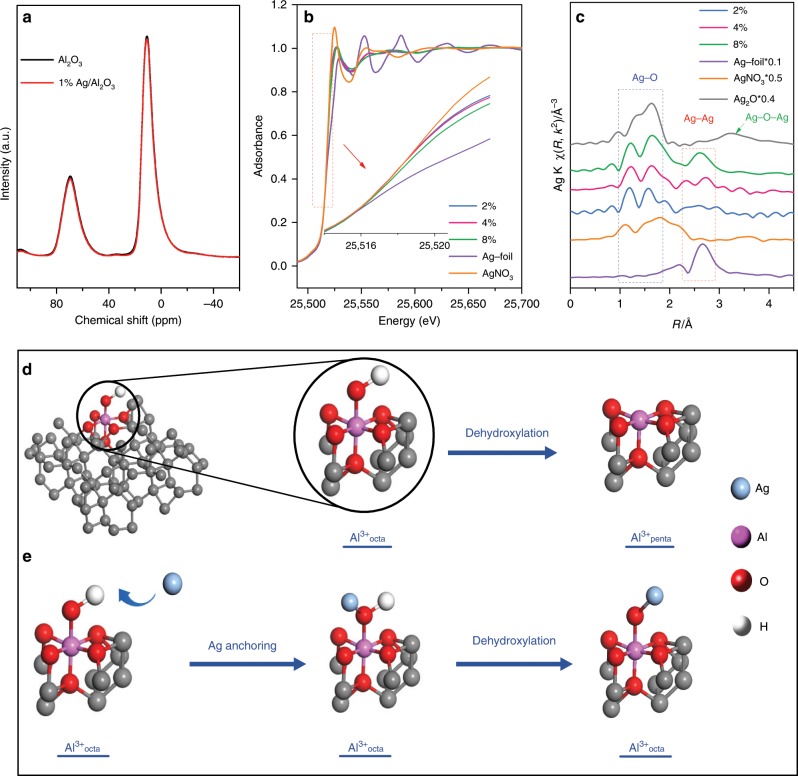
^27^Al NMR and XAFS characterization of Ag/nano-Al_2_O_3_ and analysis of Ag anchoring. **a** Normalized ^27^Al MAS NMR spectra of nano-Al_2_O_3_ and 1 wt% Ag/nano-Al_2_O_3_. **b**, **c** Ag-K edge XANES and EXAFS spectra of standard samples and Ag/nano-Al_2_O_3_ with different Ag loadings (2, 4, 8%) (Fourier transform *k* range 2.9-14.3 Å^−1^). **d** A diagram showing possible formation process of Al^3+^_penta_ from Al^3+^_octa_ through dehydroxylation on Al_2_O_3._ **e** A diagram showing possible process of Ag anchoring through interaction between Ag and Al–OH.

To solve the puzzle regarding the distinct dispersion behavior of Ag species on nano-Al_2_O_3_ and micro-Al_2_O_3_ sample, we first attempted to identify the anchoring site and the associated chemical mechanism for Ag species on γ-Al_2_O_3_. We noted that Kwak et al.^23^ reported that Al^3+^_penta_ centers on γ-Al_2_O_3_, created by dehydration and dehydroxylation at 573 K (see the diagram in Fig. 3d), are the anchoring sites for Pt, and that the coordinate saturation is the driving force due to the changes in NMR signal of Al^3+^_penta_ centers after Pt loading. Hence, first, we examined the possibility of Ag anchoring on Al^3+^_penta_ centers at room temperature by using ^27^Al solid state MAS NMR. The NMR spectra of the nano-Al_2_O_3_ and 1% Ag/nano-Al_2_O_3_ samples were collected and the results are presented in Fig. 3a. No NMR signal of Al^3+^_penta_ sites (at 35 ppm)^23^ on pristine nano-Al_2_O_3_ was seen, and the Ag loading on nano-Al_2_O_3_ apparently induced little changes in the coordination number of Al. These results show that the nano-Al_2_O_3_ is coordinately saturated during the preparation of Ag/nano-Al_2_O_3_ without pre-dehydroxylation; therefore, Al^3+^_penta_ centers are not the anchoring sites for Ag species, and there should be another site responsible for the Ag anchoring on γ-Al_2_O_3_ surface.

XRD results of Ag/nano-Al_2_O_3_ with various Ag loadings (see Supplementary Fig. 1) show typical diffraction peaks of γ-Al_2_O_3_ (JCPDS 02-1420), while no Ag-containing phases appear until the Ag loading reaches 4 wt%, indicating that Ag species are in high dispersion at low loadings. Previously, we measured the UV–vis profiles of Ag/nano-Al_2_O_3_ and Ag/micro-Al_2_O_3_ with various Ag loadings^12^. The UV–vis spectra of 1% Ag/nano-Al_2_O_3_ and 1% Ag/micro-Al_2_O_3_ after the Kubelka–Munk transformation are presented in Supplementary Fig. 2a. The spectra were deconvoluted into Gaussian subbands on the basis of the assignments. Next, the percentages of Ag species were calculated based on an analysis of the integrated peak areas. As shown in Supplementary Fig. 2b, the percentage of isolated Ag^+^ was more than 95% on nano-Al_2_O_3_, while about 60% of Ag species existed in the Ag_n_^δ+^ state on micro-Al_2_O_3_. These UV–vis results reveal that the Ag species on nano-Al_2_O_3_ are mainly the isolated Ag^+^ ions at low Ag loading (1–2 wt%) and form the metallic Ag_n_ clusters at relatively high Ag loading (4–8 wt%)^6,30,32–37^, whereas even 1% Ag on micro-Al_2_O_3_ tends to form Ag_n_^δ+^ clusters, consistent with the HAADF-STEM images. XAFS measurements were then conducted to study the valence and coordination condition of Ag species on the nano-Al_2_O_3_ surface. Ag-K edge X-ray adsorption spectra of Ag/nano-Al_2_O_3_ with different Ag loadings (2, 4, and 8 wt%) were measured, together with those of Ag foil, Ag_2_O, and AgNO_3_ as references. The normalized near-edge structure (XANES) of the samples (Fig. 3b) further shows that the Ag was in the oxidized state at low Ag loadings (2 wt%) and closer to the metallic state at high Ag loadings (4 and 8 wt%)^38,39^. Fourier transforms of *k*^2^-weighted extended X-ray absorption fine structure (EXAFS) spectra of standard samples and Ag/nano-Al_2_O_3_ with different Ag loadings (2, 4, and 8 wt%) are shown in Fig. 3c. The peak at about 1.7 Å was assigned to Ag–O interactions^40,41^, and it exhibits similar peak intensities in the spectra of the three Ag/nano-Al_2_O_3_ samples. An intense peak at 2.67 Å also appeared when Ag loading was increased to 4 or 8%. This peak is similar to that of Ag foil, and was therefore attributed to Ag–Ag metallic bonding, indicating the formation of Ag clusters in the metallic state (Ag_n_^0^)^40,41^. In addition, no Ag–O–Ag shell was detected in any of the Ag/nano-Al_2_O_3_ samples.

These results above demonstrate that Ag species initially connect to Al_2_O_3_ through oxygen bridges during Ag loading, and then aggregate to form metallic Ag when the oxygen bridges are depleted with increasing the Ag loading. Hence, it is critical to find the origin of oxygen bridges in order to understand the Ag anchoring mechanism. All of Ag species are in ionic state during the impregnation step, therefore the oxygen bridge should be only originated from the γ-Al_2_O_3_ substrate. Surface oxygen atom generally exits in a form of hydroxyl groups, and it is known that γ-Al_2_O_3_ surface entails abundant and diverse surface hydroxyl groups. Since Ag^+^ has high electron affinity^42^, we speculated that Ag species are mainly anchored by the Al–OH sites through interaction between Ag^+^ and O atoms. If so, the oxygen bridges would remain unchanged after the Ag loading (as schematically shown by the diagram in Fig. 3e).

### Correlation between Ag loading and hydroxyl content

**Fig. 4:**
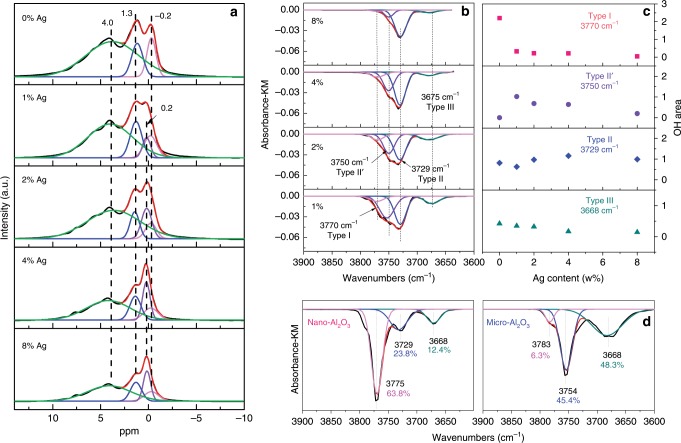
^1^H NMR and in situ DRIFTS spectra of Ag/γ-Al_2_O_3_. **a** Normalized ^1^H MAS NMR of Ag/nano-Al_2_O_3_ with different silver loadings (0, 1, 2, 4, 8%) (Samples were dehydrated at 473 K for 12 h before NMR measurements), **b** peak resolving of OH consumption peaks after in situ DRIFTS of NH_3_ adsorption over Ag/nano-Al_2_O_3_ with different silver loadings (0, 1, 2, 4, 8%), **c** relationship between Ag content and peak areas of hydroxyl groups, **d** peak resolving of OH consumption peaks after in situ DRIFTS of NH_3_ adsorption over nano-Al_2_O_3_ and micro-Al_2_O_3_.

The relationship between hydroxyl content and Ag loading was investigated through the ^1^H MAS NMR method and the results are displayed in Fig. 4a. The overlapping spectra were deconvoluted, and three peaks centered at −0.2, 1.3 and 4.0 ppm chemical shifts were fitted for the nano-Al_2_O_3_ support (zero Ag loading, 0% Ag). According to the literatures^43–46^, the bands at −0.2 and 1.3 ppm were assigned to HO-μ^1^ (Al_IV_) and HO-μ^2^ (Al_v_, Al_n_), respectively, and the broader band at 4.0 ppm was assigned to the HO-μ^3^ (Al_n_) and residual water [here, μ^1^, μ^2^, μ^3^ represent terminal (type I), doubly bridging (type II), triply bridging (type III) hydroxyls]. Loading Ag onto the nano-Al_2_O_3_ induced a substantial reduction in the peak intensity of the terminal hydroxyl groups (−0.2 ppm), evidencing the sharp decrease in the number of hydroxyl groups with 1 or 2% Ag loading. In contrast, no obvious decrease in peak intensities was observed for doubly and triply bridging hydroxyls. These results show that the terminal hydroxyl groups on Al_2_O_3_ surface are responsible for anchoring the Ag species. In addition, a new peak at around 0.2 ppm arisen with the Ag-loaded samples, thereby being relevant to the Ag species. Thus, the new peak was tentatively assigned to a new type of doubly bridging hydroxyls [type II’, HO-μ^2^ (Ag, Al_n_)], owing to the anchoring of Ag on terminal hydroxyl groups.

Surface OH groups on Al_2_O_3_ can act as acid sites, and NH_3_ adsorption could take place on these sites. Therefore, monitoring the changes in OH-related peaks by FTIR during NH_3_ adsorption can be used to characterize the types, strengths, and concentrations of surface OH groups. If Ag species occupy the OH sites when being loaded on γ-Al_2_O_3_, the intensities of negative OH peaks would decrease since fewer OH sites are available for NH_3_ adsorption. In situ DRIFTS of NH_3_ adsorption was then carried out on Ag/nano-Al_2_O_3_ samples to further study more detailed relationship between Ag anchoring and surface hydroxyl groups (see Supplementary Fig. 3). The bands at 1689, 1478, and 1395 cm^−1^ were attributed to the vibrations of NH_4_^+^ chemisorbed on Brønsted acid sites^47–49^. The bands at 1614 and 1239 cm^−1^ were assigned to NH_3_ on Lewis acid sites^49–51^. It is well established that γ-A1_2_O_3_ surfaces contain various types of OH groups, characterized by their O–H stretching frequencies. The negative bands at 3775, 3729, and 3668 cm^−1^ were assigned to the occupation of isolated hydroxyl group of type I, II, and III^52–60^, respectively. The peak intensities for the surface OH groups^52,53^ clearly decreased after Ag species were loaded, indicating that some OH sites were occupied by Ag species.

The overlapping peaks related to OH occupation over Ag/nano-Al_2_O_3_ with different silver loadings (0, 1, 2, 4, 8%) were deconvoluted (see Fig. 4b, d). Four subpeaks were assigned to three types of OH groups (type I, type II, type III) and the newly formed OH groups (type II′), respectively. The peak areas were calculated by integration, and the correlations between Ag content and hydroxyl peak areas were next analyzed. As shown in Fig. 4c, the peak area of terminal OH groups (type I) was sharply dropped at Ag loading of 1 and 2%, but further increasing the Ag content to 4 and 8% induced no obvious change in peak area. Meanwhile, a new type of doubly bridging OH groups (type II′) appeared at 3750 cm^−1^ after Ag was loaded. In contrast, there was no correlation between Ag loading and the other two types of OH groups (type II, III). These results strongly support that Ag species are anchored on the Al_2_O_3_ surface through interaction between Ag and terminal hydroxyl groups (type I), while producing a new type of hydroxyl group.

We also measured the concentration of surface OH groups on microsize Al_2_O_3_ using in situ DRIFTS spectra of NH_3_ adsorption, and compared with those on nano-Al_2_O_3_. As shown in Fig. 4d, the content of terminal hydroxyl groups in micro-Al_2_O_3_ was only 6.3 %, about ten times lower than that in nano-Al_2_O_3_ (63.8%). Since the nano-Al_2_O_3_ contains abundant terminal hydroxyls for Ag anchoring, 1% Ag can be dispersed in single-atom Ag fashion on nano-Al_2_O_3_. In contrast, there are insufficient number of terminal hydroxyl groups on micro-Al_2_O_3_ for anchoring 1% Ag. As such, Ag species aggregate into Ag cluster on micro-Al_2_O_3_.

### Anchoring mechanism of Ag on terminal hydroxyl groups

DFT calculations were carried out to investigate the interaction between Ag species and hydroxyl groups. Previous studies have shown that the (110) surface dominates on γ-Al_2_O_3_, which occupies about 70% of the total area, followed by the (100) surface (∼20% of the total area)^57,61^. The relaxed structures of the dehydrated (100) and (110) surfaces of γ-Al_2_O_3_ were shown in Supplementary Fig. 4. A total of 16 Al atoms and 24 O atoms were exposed on (110) surfaces of 2 × 2 γ-Al_2_O_3_, where Al atoms were classified as Al_trip_ and Al_tetra_, and O atoms were O_two_ and O_trip_, respectively. A total of 16 Al atoms and 24 O atoms were also exposed on the (100) surfaces of 2 × 2 γ-Al_2_O_3_. However, Al atoms were Al_tetra_ and Al_penta_, and O atoms were only O_trip_, respectively. Since our catalysts were prepared by using the impregnation method in DI-water, the (100) and (110) surfaces of γ-Al_2_O_3_ in the calculation were considered to be 100% hydroxylated. The hydroxyl surface coverage of γ-Al_2_O_3_ (100) and (110) facet in this work is 16.5 OH nm^−2^ and 14.7 OH nm^−2^, respectively. These results are within the range of OH surface coverage reported by Digne et al.^61^. We further calculated the stretching frequencies of the OH species on the (100) and (110) surfaces. The results are given in Supplementary Table 1. It is shown that the calculated frequencies are in good agreement with the data previously reported^61^, and also in line with the assignment of the experimental values (Fig. 4b).

**Fig. 5:**
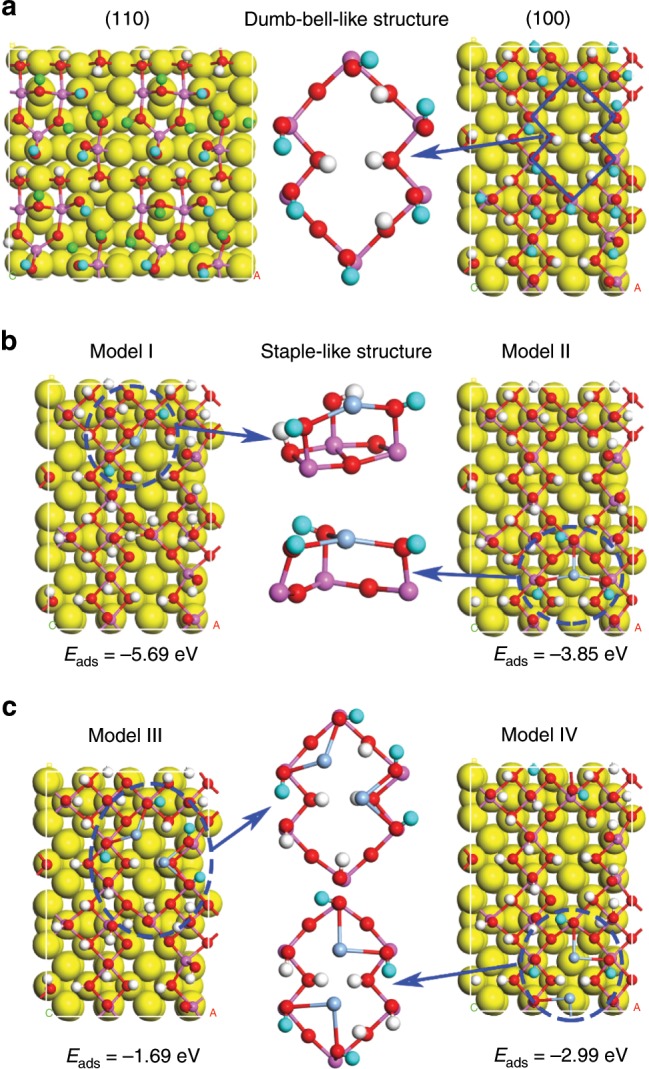
DFT calculations on interaction between Ag species and hydroxyl groups. **a** Optimized periodic models of hydroxylation Al_2_O_3_ (110) surface and Al_2_O_3_ (100) surface; **b** Optimized periodic models (model I and model II) of the adsorption of Ag atom on (100) surface of Al_2_O_3_; **c** Optimized periodic models (model III and model IV) of the adsorption of two Ag atoms on (100) surface of Al_2_O_3_. (pink: Al atom, red: O atom, blue: H atom in terminal hydroxyls (type I), green: H atom in doubly bridging hydroxyls (type II), white: H atom in triply bridging hydroxyls (type III)).

The hydroxylated (110) and (100) surfaces of γ-Al_2_O_3_ were then relaxed (see Fig. 5a). For the (110) surface, there are three types of hydroxyl groups (types I–III), and the fraction of terminal hydroxyl groups (type I) is ~30%. For the (100) surface, surface reconstruction occurs after hydroxylation, and all Al atoms become six-coordinated. So, only two types of hydroxyl groups (type I and type III) are present on the (100) surface, and the fraction of terminal hydroxyl groups (type I) is 52%. These results indicate that terminal hydroxyl groups (type I) are more easily formed on (100) surfaces of γ-Al_2_O_3_. XRD profiles (Supplementary Fig. 5) show that nano-γ-Al_2_O_3_ exhibited more (100) surfaces than micro-γ-Al_2_O_3_, supporting that nano-γ-Al_2_O_3_ entails much more terminal hydroxyl groups (type I) (see Fig. 4).

We then calculated the interaction between Ag atom and different types of hydroxyls on the (110) and (100) surfaces of γ-Al_2_O_3_. For the (110) surface, considering different coordination environments of hydroxyl groups, there are three types of terminal hydroxyls (type I), two types of doubly bridging hydroxyls (type II), and one type of triply bridging hydroxyls (type III). The relaxed structure of Ag binding on the hydroxylated (110) surfaces of γ-Al_2_O_3_ are presented in Supplementary Fig. 6. It is shown that Ag species cannot be stably bonded to any sites, demonstrating that the (110) surface of γ-Al_2_O_3_ cannot anchor Ag species.

For the (100) surface, only terminal hydroxyls (type I) are on the surface. A stable dumb-bell-like double diamond structure with six terminal hydroxyl groups was formed on the (100) surface (Fig. 5a, blue dotted lines). The relaxed structure of Ag binding on the (100) surfaces of γ-Al_2_O_3_ is shown in Fig. 5b. Despite the Ag atom is adjacent to O atom of terminal hydroxyls (type I) or triply bridging hydroxyls (type III) in the initial model, the optimized models show that Ag can be only stably bonded to terminal hydroxyls (type I), and a staple-like structure was formed by consuming two or three terminal hydroxyls (type I), producing a new type of doubly bridging hydroxyls (type II′) (consistent with NMR and DRIFTS results shown in Fig. 4). The adsorption energies of Ag in the model I and II are −5.69 and −3.85 eV, respectively, indicating that Ag species are strongly anchored on terminal hydroxyls (type I).

The relaxed structures of second Ag atom binding on the (100) surfaces of γ-Al_2_O_3_ are shown in Fig. 5c. The second Ag atom was still bonded to terminal hydroxyls (type I), and the adsorption energies of Ag in the model III and model IV are −1.69 and −2.99 eV, respectively. These results indicate that when Ag loading is low and sufficient terminal hydroxyl groups are available, Ag species are mainly anchored on the terminal hydroxyl group sites and easily form a stable single-atom Ag dispersion. However, when Ag loading is high, all terminal hydroxyls are consumed, Ag no longer can be anchored at hydroxyl sites, and it then easily agglomerates and forms metallic Ag cluster during high temperature calcination.

Kwak et al. have suggested that Al^3+^ (Al^3+^_penta_) centers are the anchoring sites for Pt species on γ-Al_2_O_3_^23^. Based on the present results, we propose that Pt anchoring on the γ-Al_2_O_3_ might be also closely related to the terminal hydroxyl groups rather than Al^3+^_penta_. We measured and compared the ^27^Al MAS NMR signals of nano-Al_2_O_3_ pretreated with different conditions. The results are presented in Supplementary Fig. 7. When γ-Al_2_O_3_ was dehydroxylated at 673 K for 12 h, the NMR signal of Al^3+^_penta_ was clearly detected. However, after the dehydroxylated nano-Al_2_O_3_ was exposed to ambient air for 12 h, or impregnated in DI-water for 2 h followed by dryness at 378 K for 6 h, the NMR signal of Al^3+^_penta_ almost disappeared, indicating that Al^3+^_penta_ sites can be easily coordinatively saturated by interaction with H_2_O. These results demonstrate that Al^3+^_penta_ sites are unlikely available on γ-Al_2_O_3_ for Ag/Pt anchoring during the catalyst preparation by using impregnation method (in DI-water) whether γ-Al_2_O_3_ was pretreated or not. We also calculated the adsorption of Ag/Pt atoms on the Al^3+^_penta_ centers. As shown in Supplementary Figs. 8 and 9, both Ag and Pt atoms can only coordinate with two O atoms adjacent to Al^3+^_penta_ but not directly with Al^3+^_penta_ sites. The calculated adsorption energies of Ag/Pt on O atoms adjacent to Al^3+^_penta_ were also much weaker than the adsorption energies of Ag/Pt on the terminal hydroxyl groups, indicating that both Ag and Pt atoms tend to interact with terminal hydroxyl groups even if the Al^3+^_penta_ center is available on γ-Al_2_O_3_. Thus, we believe that the terminal hydroxyl groups are also responsible for the anchoring of Pt on γ-Al_2_O_3_.

In summary, this comprehensive study reveals that, other than electronic defects and Al^3+^_penta_ centers, the hydroxyl group is the key site for metal–support interactions that affects the valence state, morphology, and dispersion of Ag species on γ-Al_2_O_3_. Based on the measurements of MAS NMR, in situ DRIFTS, HAADF-STEM, we show that the Ag species are mainly anchored by the terminal hydroxyl groups (type I) on γ-Al_2_O_3_ through interaction between Ag and the O atom in Al-OH groups. Nanosized γ-Al_2_O_3_ entails abundant terminal hydroxyl groups so that Ag could be atomically dispersed on nano-Al_2_O_3_, while Ag tends to agglomerate to form the Ag clusters on micro-Al_2_O_3_ since the number of terminal hydroxyl groups are insufficient for Ag binding. Single-atom Ag dispersion on Ag/nano-Al_2_O_3_ gives rise to markedly higher catalytic performance in HC-SCR of NO, compared with Ag-cluster dispersion. DFT calculations confirm that it is the (100) surfaces of γ-Al_2_O_3_ that can accommodate much more terminal hydroxyl groups than the (110) surfaces. As such, single Ag atom can be only anchored by the terminal hydroxyls on the (100) surfaces through consuming two or three terminal hydroxyls and forming new doubly bridging hydroxyls. Our study resolves the puzzle on why the single-atom Ag dispersion can be spontaneously achieved only on surface of nanosized γ-Al_2_O_3_ since nanosized γ-Al_2_O_3_ entails predominantly the (100) surfaces.

## Methods

### HAADF-STEM

High-angle annular dark-field scanning transmission electron microscopy (HAADF-STEM) was performed on a Cs-corrected JEOL JEM-ARM 200F operated at 200 kV.

### Activity test

The activity test for HC-SCR was performed at steady state in a fixed-bed flow reactor with the gas hourly space velocity (GHSV) of 100,000 h^−1^. The mixture gas of C_3_H_6_–H_2_-SCR is NO 800 ppm, C_3_H_6_ 1565 ppm, H_2_ 1%, O_2_ 10%, and N_2_ balance. The mixture gas of ethanol-SCR is NO 800 ppm, C_2_H_5_OH 1565 ppm, O_2_ 10%, H_2_O 5%, and N_2_ balance. An online FTIR spectrometer was used to continuously analyze the concentrations of NO_*x*_.

### MAS NMR

^27^Al and ^1^H NMR spectra were recorded at 11.7T on a Bruker-Advance^III^ 500 spectrometer equipped with a 4 mm double-resonance probe. The corresponding resonance frequency and magic angle spinning rate were 500.57 MHz and 10 kHz respectively. ^27^Al NMR measurements were conducted with no pretreatment on the samples. For ^1^H NMR measurements, samples were pre-dehydrated on a vacuum line. The temperature was gradually increased at a rate of 1 K min^−1^ and the sample was kept at a final temperature of 473 K and at a pressure below 10^−3^ Pa for 12 h. The average weight of samples is 0.0513 g and the deviation for samples is lower than 2.7%, all NMR spectra were normalized to the sample weight.

### XAFS

Ag K-edge XAFS spectra were recorded in transmission mode at room temperature, using the BL14W1 XAFS beam line at the Shanghai Synchrotron Radiation Facility. The XANES and EXAFS data reduction and analysis were performed using the Athena program that is part of the IFFEFIT software package^62,63^. The filtered *k*^2^ weighted *χ*(*k*) was Fourier-transformed into R space (*k* range: 2.9–14.3 Å^−1^ for Ag-K EXAFS).

### In situ DRIFTS

The in situ diffuse reflectance infrared Fourier transform spectroscopy (DRIFTS) was conducted on a Nexus 670 (Thermo Nicolet) FTIR equipped with an MCT/A detector. All spectra were recorded in the range 4000–800 cm^−1^ by accumulating 100 scans with a resolution of 4 cm^−1^. A background spectrum was subtracted from each spectrum. Samples were pretreated at 473 K for 30 min in a flow of N_2_ + O_2_ (20%) before in situ DRIFTS measurements.

### DFT calculations

Geometries and energies were calculated using the DFT method in the formalism of Perdew–Burke–Ernzerhof (PBE) functional^64^ with van der Waals correction proposed by Becke-Jonson (i.e., DFT-D3 method)^65^, as implemented in the Vienna ab initio simulation package (VASP 5.4.4)^66^. The projector augmented wave method was used to describe the interaction between the ions and the electrons^67^. Convergence tests were performed for all initial parameters. The vacuum gap was 20 Å so that inter-slab interactions were negligible in the periodic systems. DFT calculations were carried out with a plane-wave energy cutoff of 400 eV. Based on our previous reports^68,69^, the dehydrated (110) and (100) surfaces of γ-Al_2_O_3_ were modeled using (2 × 2) supercells. According to our preliminary convergence test, Monkhorst−Pack k-point sets of (1 × 1 × 1) were used for the Al_2_O_3_ surfaces. The top two layers and the adsorbents were fully relaxed, while the bottom layers were fixed to mimic the bulk region. The adsorption energies of Ag/Pt on the γ-Al_2_O_3_ surface were calculated as follows: *E*_ad_ = *E*_adsorbate + surface_ ‒ (*E*_surface_ + *E*_adsorbate_), where *E*_adsorbate+surface_ and *E*_surface_ are the total energies of the adsorbed system and alumina slab, respectively; and *E*_ad_ reflects the stability of the adsorbates on the γ-Al_2_O_3_ surface. Negative *E*_ad_ values mean that the adsorbed state is energetically favorable. The DFT-predicted adsorption energies of Ag/Pt atom are with respect to the energy of a gas-phase Ag atom. All the DFT computations were spin polarized. We compute the adsorption energy of Ag on the γ-Al_2_O_3_ (110) surface, with (−5.76 eV) and without dipole correction (−5.69 eV). The difference in the adsorption energy of Ag is less than 1.2%. Consequently, all calculation results do not include the dipole correction.

## Supplementary information

Supplementary Information

## Data Availability

The authors declare that the data supporting the findings of this study are available within the paper and its supplementary information files. All relevant data are available from the correspondence authors upon reasonable request.
