# Supplementary material for: Resolving the puzzle of single-atom silver dispersion on nanosized γ-Al2O3 surface for high catalytic performance
Source: Nat Commun. 2020 Jan 27;11:529. doi: 10.1038/s41467-019-13937-1 (PMC6985108; doi:10.1038/s41467-019-13937-1)
Supplement: Supplementary file 1 — Supplementary Information [file 41467_2019_13937_MOESM1_ESM.pdf]

SUPPLEMENTARY INFORMATION

Resolving the puzzle of single-atom silver dispersion on nano-sized γ-Al_2O_3 surface for high catalytic performance

Fei Wang *et al.*

Supplementary Methods:

Preparation of catalyst. Nano sized γ-Al_2O_3 (nano-Al_2O_3, Aladdin, average size 10 nm, 216.1 m^2 g^{-1}) supported Ag (Ag/nano-Al_2O_3) catalysts with different Ag loadings (1, 2, 4, 6 and 8 wt.%) and micro sized γ-Al_2O_3 (micro-Al_2O_3, Sigma-Aldrich, average size 5 μm, 222.6 m^2 g^{-1}) supported Ag catalyst (1% Ag/micro-Al_2O_3) were prepared by an impregnation method. An appropriate amount of γ-Al_2O_3 was added into aqueous solutions of silver nitrate ($AgNO_3$, Alfa Aesar) with different concentrations. After stirring for 2 h, the excess water was removed by vacuum rotary evaporation. The samples were dried at 105 °C overnight and then calcined at 500 °C for 3 h in air. The catalysts were sieved into 40-60 mesh powders before testing.

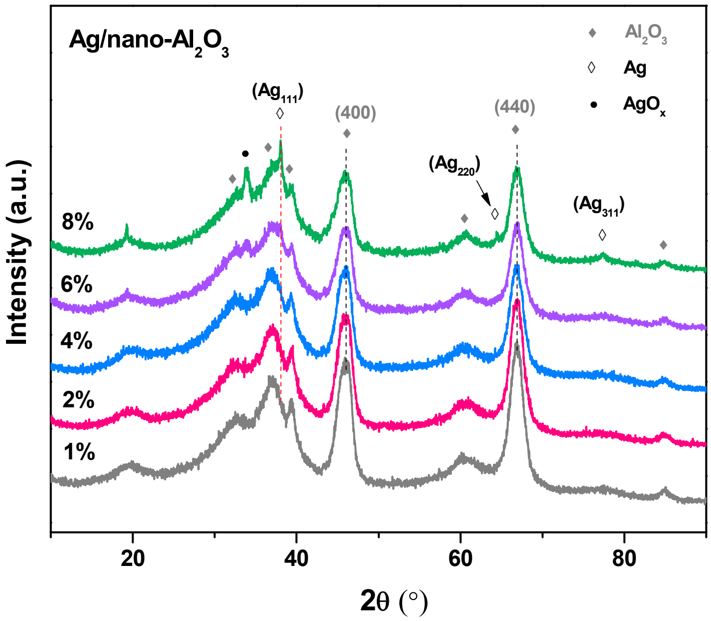

Supplementary Figure 1. XRD profiles of Ag/nano-Al_2O_3 with different Ag loadings.

The XRD patterns of the samples with different Ag loadings (1-8 wt.%) are shown in Supplementary Figure 1. The diffraction patterns of the samples were quite similar and they all showed diffraction peaks at 31.5°, 37.5°, 39.3°, 45.7°, 60.5°, 66.6° and 84.5° corresponding to (220), (311), (222), (400), (511), (440) and (444) of γ-Al_2O_3 [1] (JCPDS 02-1420), respectively. The diffraction peaks of Ag-containing phases could hardly be identified until the Ag loading reached 4 wt%, and their intensities became stronger with increasing Ag content for samples 6% Ag/nano-Al_2O_3 and 8% Ag/nano-Al_2O_3. The diffraction peaks at 38.1°, 64.5° and 77.4° corresponded to the (111), (220), and (311) lattice planes of Ag metal [2, 3] (JCPDS 87-0717). A diffraction peak of Ag_2O could also be identified on 6% Ag/nano-Al_2O_3 and 8% Ag/nano-Al_2O_3 at 33.7° [4] (JCPDS 41-1104). These results confirm that the crystallite sizes of Ag phases (Ag/Ag_2O) increased with increasing Ag loading.

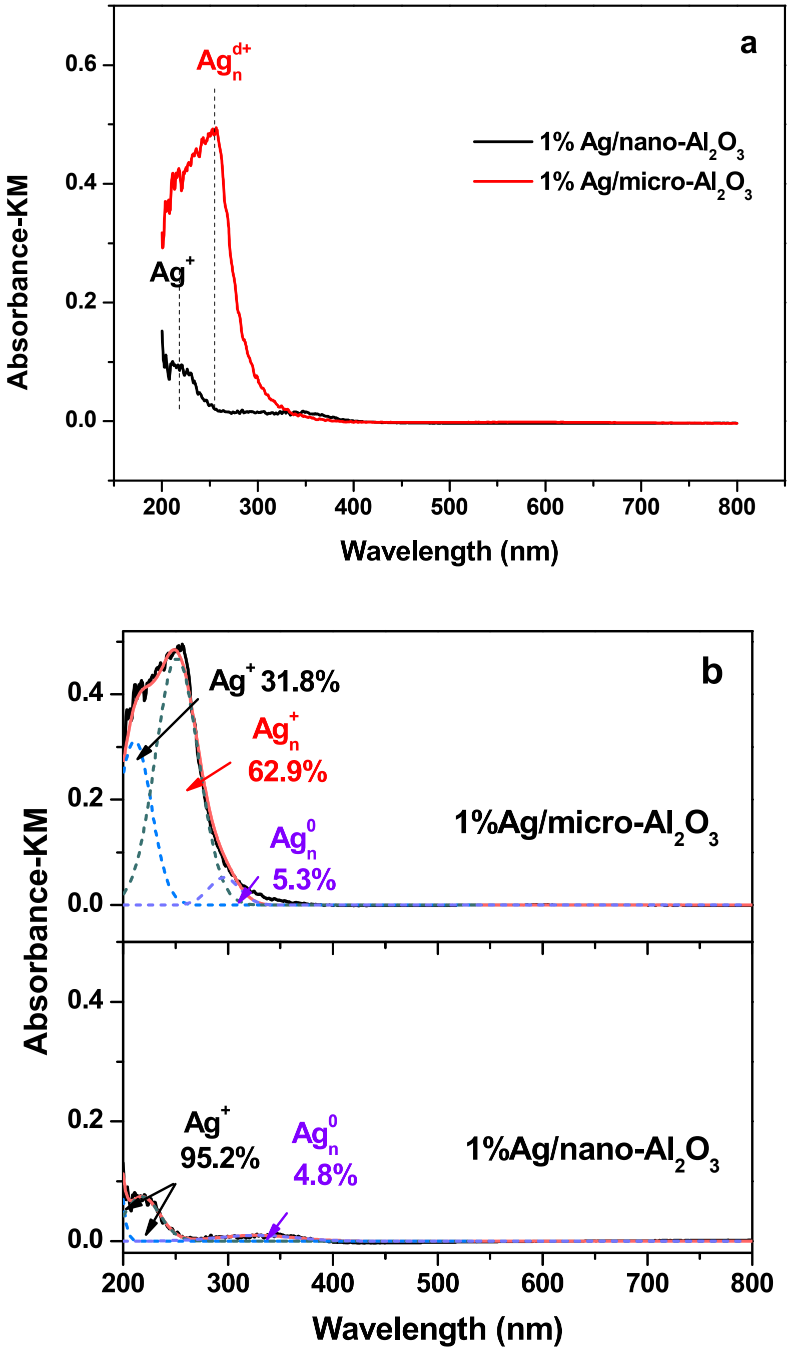

Supplementary Figure 2. UV-vis profiles (a) and deconvoluted UV-vis profiles (b) of 1% Ag/nano-Al_2O_3 and 1% Ag/micro-Al_2O_3 after the application of the Kubelka–Munk function.

UV-vis spectra show that 1% Ag tends to form clusters on micro-Al_2O_3 while highly dispersed on nano-Al_2O_3 [5, 6].

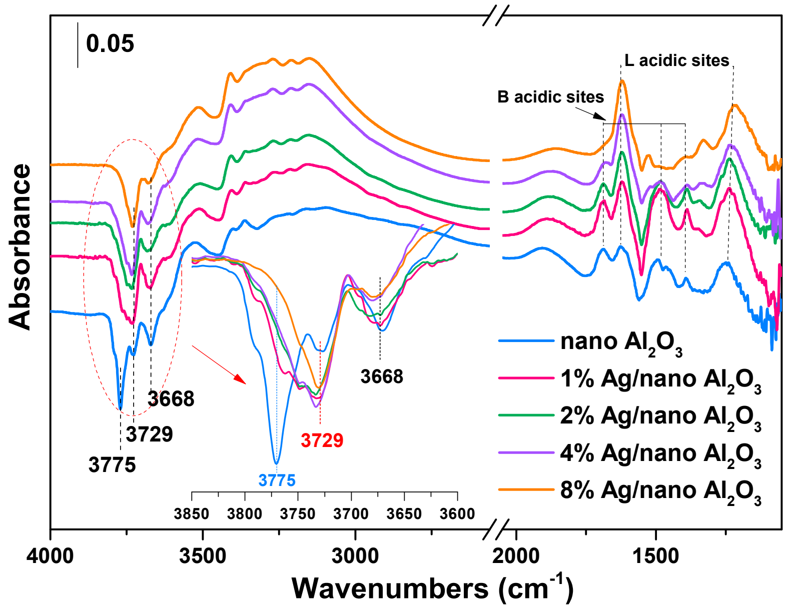

Supplementary Figure 3. In situ DRIFTS results of NH_3 adsorption over Ag/nano-Al_2O_3 with different Ag loadings (0, 1, 2, 4, 8%) at room temperature

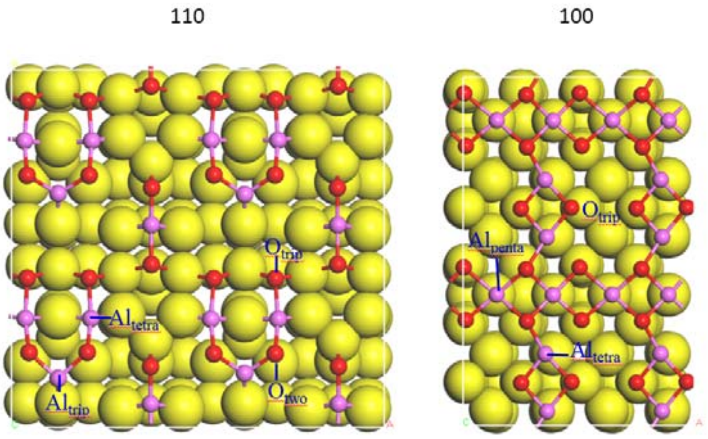

Supplementary Figure 4. Optimized periodic models of γ-Al_2O_3 surface. (a) Al_2O_3 (110) surface and (b) Al_2O_3 (100) surface (pink: Al atom, red: O atom).

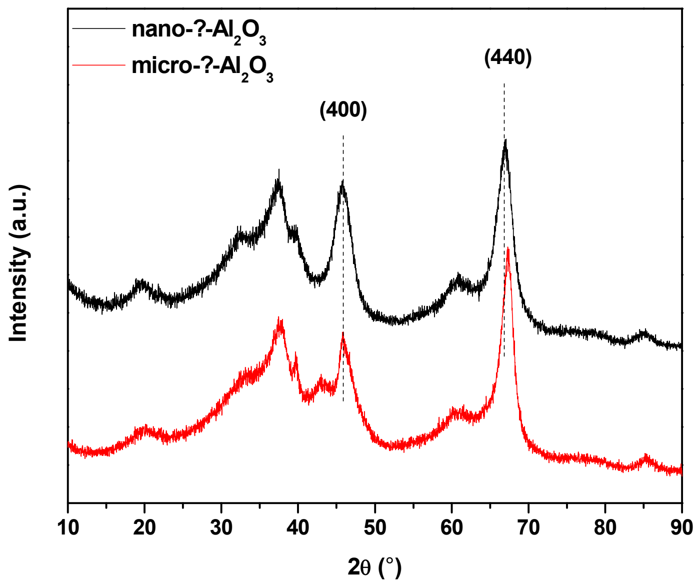

Supplementary Figure 5. XRD profiles of nano-γ-Al_2O_3 and micro-γ-Al_2O_3.

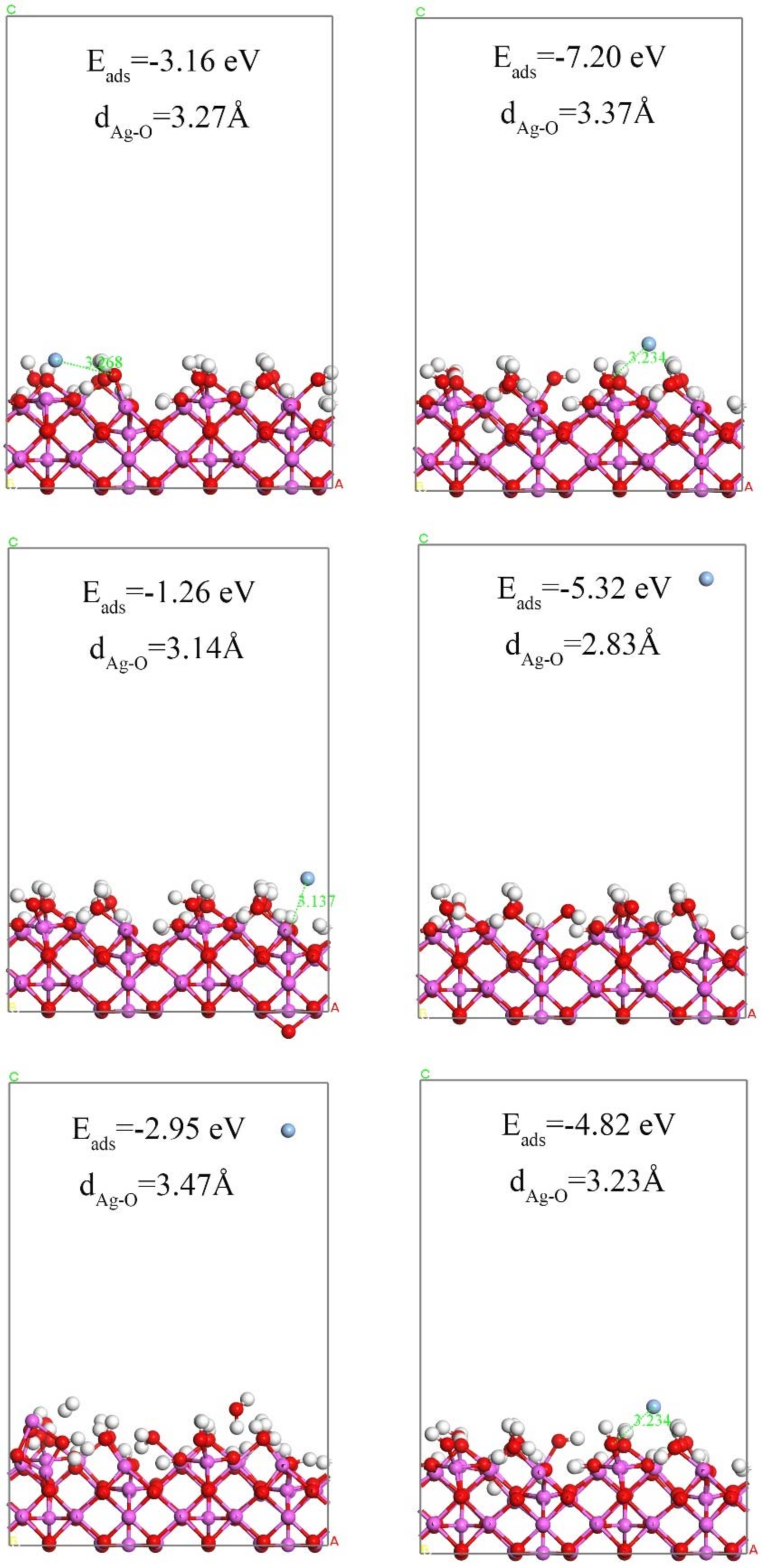

Supplementary Figure 6. Optimized periodic models of the adsorption of Ag on different types of hydroxyls on the (110) surface of γ-Al_2O_3 (pink: Al atom, red: O atom, white: H atom, indigo: Ag atom).

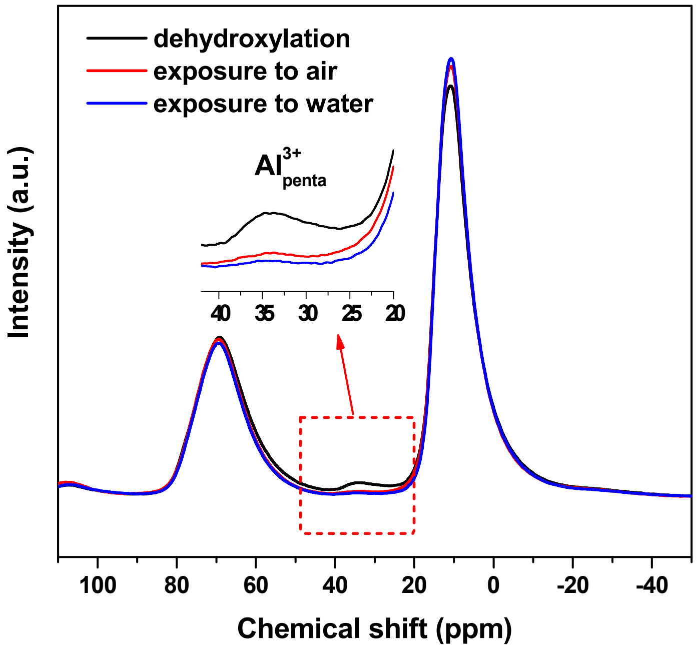

Supplementary Figure 7. Normalized ^{27}Al MAS NMR spectra of nano-Al_2O_3 pretreated with different conditions. Sample 1 (dehydroxylation): Al_2O_3 was pretreated at 673 K at a pressure below 10^{-3} Pa for 12 h before NMR measurements. Sample 2 (exposure to air): Al_2O_3 was first pretreated at 673 K for 12 h and then exposed to ambient air at room temperature for 12 h. Sample 3 (exposure to water): Al_2O_3 was first pretreated at 673 K for 12 h and then impregnated in DI-water for 2 h, followed by dryness at 378 K for 6 h in air.

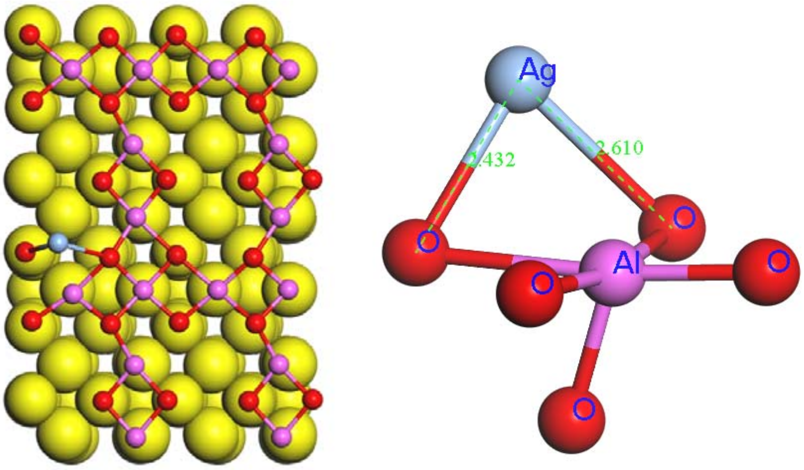

Supplementary Figure 8. Optimized structure of the adsorption of Ag atom on the Al^{3+}_{penta} centers of γ-Al_2O_3

The adsorption of Ag atoms on the Al^{3+}_{penta} centers was studied. As shown in Supplementary Figure 8, Ag atoms can be only coordinated with two O atoms adjacent to Al^{3+}_{penta} but not directly with Al^{3+}_{penta}. The calculated adsorption energy is -0.70 eV, much weaker than the adsorption energy of Ag on the terminal hydroxyl groups (-3.85~-5.69 eV), indicating that Ag atoms tend to interact with terminal hydroxyl groups even if the Al^{3+}_{penta} center is available on γ-Al_2O_3.

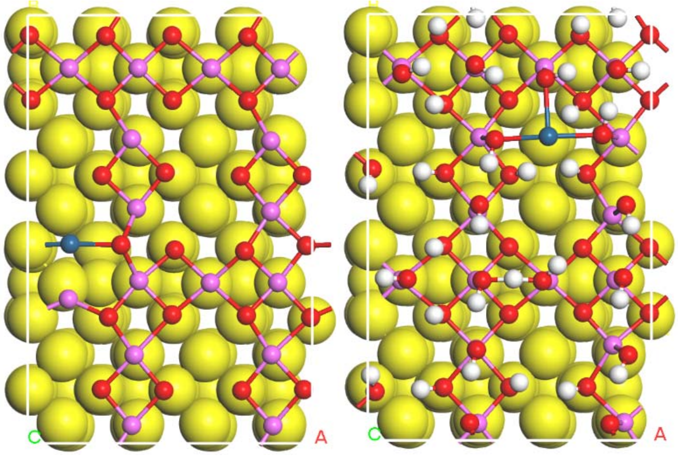

Supplementary Figure 9. Optimized structure of the adsorption of Pt atom on the Al^{3+}_{penta} centers and the terminal hydroxyl groups of γ-Al_2O_3

The adsorption of Pt atoms on the Al^{3+}_{penta} centers and the terminal hydroxyl groups is studied. As shown in Supplementary Figure 9, Pt atoms can be only connected with two O atoms adjacent to Al^{3+} but no directly with Al^{3+}_{penta}. The calculated adsorption energy is -1.61 eV, much weaker than the adsorption energy of Pt on the terminal hydroxyl groups (-3.40 eV), indicating that Pt atoms also tend to interact with terminal hydroxyl groups even if the Al^{3+}_{penta} center is available on γ-Al_2O_3.

Supplementary Table 1. Computed vibrational stretching frequencies of surface hydroxyl groups on γ-Al_2O_3

models	surface	d O-H(Å)	$V_{cal.}$(cm^{-1})	$V_{cal.}$(cm^{-1}) [a]	$V_{exp.}$(cm^{-1})
terminal hydroxyls	(100)	0.970	3716	3796	3775
triply bridging hydroxyls	(100)	0.977	3433	3513	3668
terminal hydroxyls-1	(110)	0.957	3765	3845	3775
terminal hydroxyls-2	(110)	0.961	3646	3726	
terminal hydroxyls-3	(110)	0.962	3711	3791	
doubly bridging hydroxyls-1	(110)	0.965	3732	3812	3729
doubly bridging hydroxyls-2	(110)	0.963	3168	3247	
doubly bridging hydroxyls-3	(110)	0.992	3184	3264	
doubly bridging hydroxyls-4	(110)	1.001	3192	3272	
triply bridging hydroxyls	(110)	0.971	3506	3586	3668
triply bridging hydroxyls	(110)	1.004	2935	3015	

[a] The frequencies are corrected by an anharmonicity term of 80 cm^{-1} (see *Journal of Catalysis* 211, 1–5 (2002) [7])

Supplementary References:

1. Li, M., Li, H., Jiang, F., Chu, Y. & Nie, H. Effect of surface characteristics of different alumina on metal–support interaction and hydrodesulfurization activity. *Fuel* **88**, 1281-1285 (2009).
2. Savel'eva, A.S. & Vodyankina, O.V. Formation of the active surface of Ag/SiO_2 catalysts in the presence of FeOx additives. *Russian Journal of Physical Chemistry A* **88**, 2203-2208 (2014).
3. Wang, F. et al. Nanosize effect of Al_2O_3 in Ag/Al_2O_3 catalyst for the selective catalytic oxidation of ammonia. *ACS Catal.* **8**, 2670-2682 (2018).
4. Doronkin, D.E., Fogel, S., Gabrielsson, P., Grunwaldt, J.-D. & Dahl, S. Ti and Si doping as a way to increase low temperature activity of sulfated Ag/Al_2O_3 in H_2-assisted NH_3-SCR of NO_x. *Appl. Catal. B* **148-149**, 62-69 (2014).
5. Shibata, J. et al. Structure of active Ag clusters in Ag zeolites for SCR of NO by propane in the presence of hydrogen. *J. Catal.* **227**, 367-374 (2004).
6. Kim, P.S., Kim, M.K., Cho, B.K., Nam, I.-S. & Oh, S.H. Effect of H_2 on deNOx performance of HC-SCR over Ag/Al_2O_3: Morphological, chemical, and kinetic changes. *J. Catal.* **301**, 65-76 (2013).
7. Digne, M., Sautet, P., Raybaud, P., Euzen, P. & Toulhoat, H. Hydroxyl Groups on γ-Alumina Surfaces: A DFT Study. *J. Catal.* **211**, 1-5 (2002).
